# Melatonin reduces the severity of experimental amoebiasis

**DOI:** 10.1186/1756-3305-4-62

**Published:** 2011-04-18

**Authors:** Aline C França-Botelho, Juliana L França, Fabrício MS Oliveira, Eduardo L Franca, Adenilda C Honório-França, Marcelo V Caliari, Maria A Gomes

**Affiliations:** 1 Department of Parasitology, Institute of Biological Sciences, Universidade Federal de Minas Gerais, Belo Horizonte, Minas Gerais, Brazil; 2 Institute of Health Sciences, Centro Universitário do Planalto de Araxá, Araxá, Minas Gerais, Brazil; 3 Department of Biochemistry, Faculty of Medicine, Universidade de São Paulo, Ribeirão Preto, São Paulo, Brazil; 4 Department of General Pathology, Institute of Biological Sciences, Universidade Federal de Minas Gerais, Belo Horizonte, Minas Gerais, Brazil; 5 Institute of Biological Sciences and Health, Universidade Federal do Mato Grosso, Pontal do Araguaia, Mato Grosso, Brazil

## Abstract

**Background:**

Melatonin has immunomodulatory effects but very little is known about its influence in protozoan infections, such as *Entamoeba histolytica*, which causes amoebiasis, a disease with significant morbidity and mortality. In this study, we evaluated the effects of exogenous melatonin interference in experimental amoebiasis and on interactions between human blood cells and *E. histolytica *trophozoites.

**Methods:**

The effect of melatonin was investigated in models of experimental amoebiasis in hamsters and rats by evaluating the area of necrosis induced by *E. histolytica*. The activity of melatonin on the interactions between leukocytes and amoebae was determined by examining leukophagocytosis. For *in vitro *tests, polymorphonuclear and mononuclear human blood leucocytes were incubated with *E. histolytica *trophozoites.

**Results:**

The areas of amoebic necrosis were significantly reduced in animals treated with melatonin. Melatonin treatment increased leukophagocytosis but was associated with a greater number of dead amoebae.

**Conclusions:**

These results suggest that melatonin may play a beneficial role in the control of amoebic lesions, raising the possibility that this drug may be used as an adjuvant in anti-amoebic therapy.

## Background

Melatonin [N-acetyl-5-methoxytryptamine] is an indoleamine synthesised from tryptophan. The physiological properties of melatonin are not limited to its neuroendocrine role in controlling circadian rhythms [1]; several other actions have been discovered. Melatonin has been shown to increase innate and acquired immunity [2], to activate the bone marrow and lymph nodes [3], to enhance NK cell activity [4,5] and antibody-dependent cell cytotoxicity [6], to increase T cell proliferation *in vivo *and *in vitro *[7,8] and to activate monocytes [9,10] and neutrophils [11].

Melatonin can stimulate innate immune cells, primarily leukocytes, which represents an important anti-bacterial mechanism [12,13]; however, very little is known about its influence on protozoan infections.

*Entamoeba histolytica *is an enteric protozoan parasite that infects 500 million people, causes amoebiasis in 50 million and kills 100 000 individuals annually, thus constituting a serious health public problem [14]. The disease is widely distributed worldwide, but its incidence is highest in places with insufficient basic sanitation. Several aspects of this host parasite relationship, such as parasite virulence and host susceptibility, are poorly understood. The course of infection begins with an inflammatory process that recruits eosinophils, lymphocytes, neutrophils and macrophages [15]; however, despite this immune cell recruitment, tissue destruction progresses, generating a typical amoebic necrotic area.

Melatonin has been reported to have immunomodulatory effects in the cases of toxoplasmosis [16-18], malaria [19,20] and Chagas disease [21].

One of these studies investigated the effect of melatonin and zinc on the immune response to *Toxoplasma gondii *retinochoroiditis in the rat model (pinealectomy or not) of infection and to establish the possible value of supplementation as adjunctive therapeutic agents in the treatment of *T. gondii *retinochoroiditis. Melatonin should be considered as an adjunctive therapy to classic treatment of *Toxoplasma *retinochoroiditis, especially in immunosuppressed and elderly patients if our data are confirmed in a clinical setting [18].

In model Chagas disease, animals treated with melatonin showed a significant reduction in the number of blood trypomastigotes during the acute phase of infection compared with untreated animals and increase in leucocytes numbers during the peak of parasitaemia [21].

Nothing has yet been reported regarding amoebiasis. This pioneering study aims to evaluate the influence of exogenous melatonin in experimental amoebic infections and its effects on amoebae-leukocyte interactions *in vitro*.

## Results

### *In vivo *results

**Figure 1:**
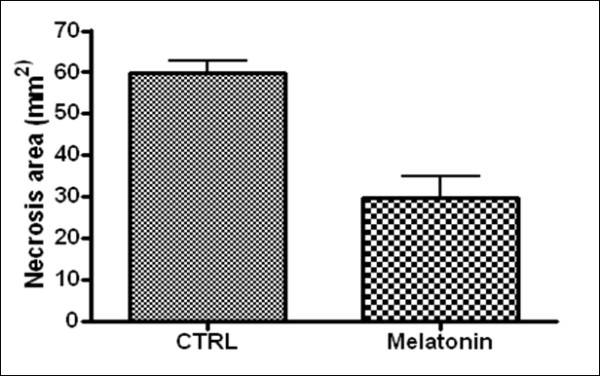
**Amoebic hepatic necrosis**. Amoebic hepatic necrosis in mm^2 ^and standard deviation in hamsters treated and not treated with melatonin.

*E. histolytica*-infected hamsters that were treated with melatonin exhibited significantly reduced areas of hepatic necrosis relative to those in the control group (p < 0.05). The necrotic areas in the melatonin-treated animals were about half the size of the lesions in the untreated animals (Figure 1). Furthermore, only 1 of the 6 rats treated with melatonin showed amoebic lesions in its caecum, whereas 5 of the 6 untreated rats had caecal lesions.

**Figure 2:**
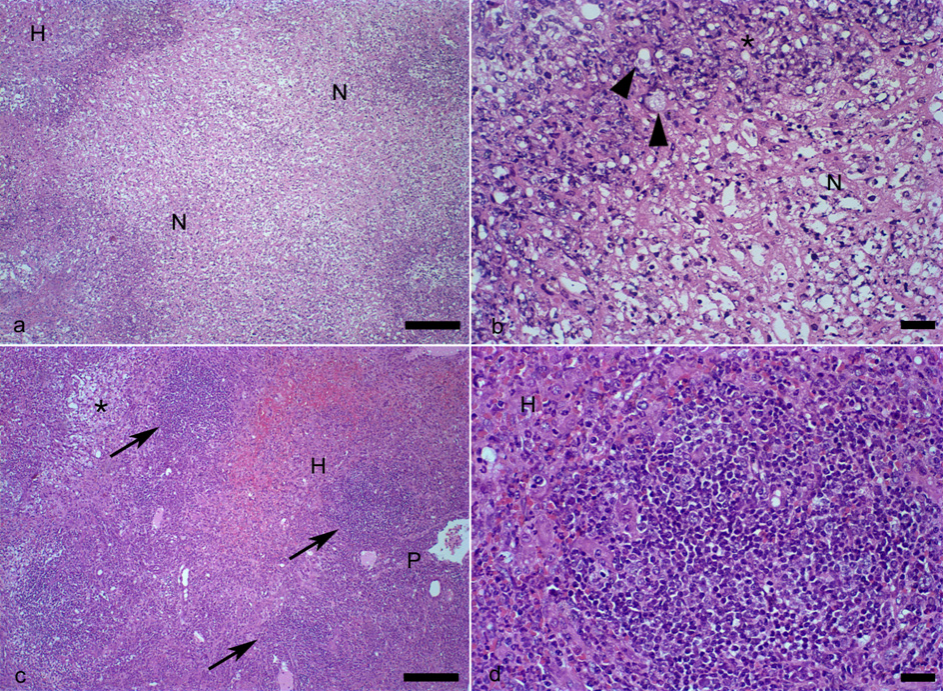
**Histology of liver from hamsters**. Histology of liver from hamsters inoculated with the HM1 strain of *E. histolytica*. (a) significant necrosis of the liver parenchyma (N) of untreated hamster melatonin. Non-necrotic hepatic parenchyma (H), (b) Detail of preceding figure showing trophozoites (arrowheads) adjacent to the large amount of cellular debris and moderate inflammatory infiltrate (*). Necrosis (N), (c) non-necrotic hepatic parenchyma (H) of hamsters that received melatonin. Note the presence of focal inflammatory infiltrate rich in mononuclear cells (arrows). Granulation tissue (*). Portal space (P), (d) Detail of previous picture showing one of the focal infiltrates predominantly composed of lymphocytes and macrophages. Non-necrotic liver parenchyma (H). H&E. (a) and (c) Bar 100 μm; (b) and (d) Bar 20 μm.

Figure 2 shows the histopathological analysis of hamsters inoculated with the HM1 strain of *E. histolytica *either without (a and b) or with melatonin treatment (c and d). The area of tissue destruction was smaller in animals that were treated with melatonin than in controls that did not receive melatonin treatment. Microscopic observation of hepatic tissues from animals that did not receive treatment showed that necrosis was often distributed over wide areas. In contrast, necrotic regions were interspersed between areas of normal tissue in animals that received melatonin treatment. The inflammatory infiltrate was more intense in the treated animals and showed focal zones comprised of large numbers of mononuclear cells and some giant cells. These focal zones were absent in animals that did not receive melatonin treatment.

### *In vitro *results

**Figure 3:**
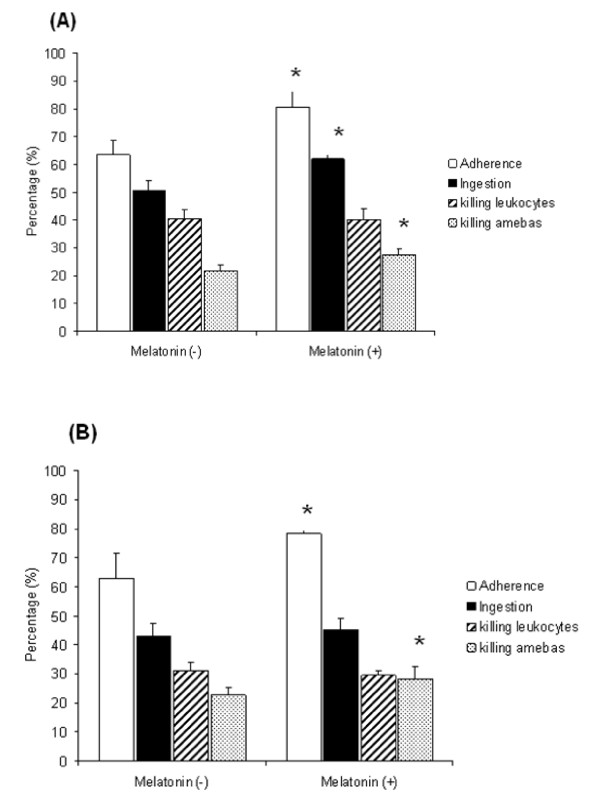
**Leukophagocytosis**. Effects of melatonin on leukophagocytosis of mononuclear (A) and polymorphonuclear (B) human leukocytes accomplished by amoebae, evaluated by the parameters: adherence, leukocytes ingestion, killing leukocytes and killing amoebae. Results are the percent means and standard deviation of 5 repetitions. Statistically different (p < 0.05) versus control (melatonin absent).

Melatonin increased the adherence of *E. histolytica *trophozoites to mononuclear (MN) and polymorphonuclear (PMN) leukocytes." The highest adherence rate (80.4%) was seen with MN cells in the presence of melatonin. In analysing the ingestion of leukocytes by the amoebae, we found that melatonin only induced a significant increase in the ingestion of PMN leukocytes: the ingestion rate was 50.6% in the absence of melatonin and 62% in its presence. We observed a significant increase in the percentage of dead amoebae during leukocyte internalization in the presence of melatonin, with the frequency of dead amoebae increasing by 26% for those ingesting MN leucocytes and 24% for those ingesting PMN leucocytes, relative to those in non-melatonin-treated cultures (Figures. 3a and 3b).

During phagocytosis, in the presence of melatonin, superoxide released from MN interacting with amoebae was 26.2 nmol and PMN interacting with amoebae was 27.8 nmol. The controls were 10.4 nmol and 16.6 nmol respectively. SOD released from MN interacting with amoebae was 108.8 and PMN interacting with amoebae was 125.6. The controls in the absence of melatonin were 76.4 and 66.6 respectively.

## Discussion

Little is known about the immunomodulatory effects of melatonin in protozoan infections. This study is the first to evaluate the action of melatonin on *E. histolytica in vivo *and *in vitro*. The results of our experimental amoebiasis studies showed a significant reduction in the areas of amoebic necrosis in response to melatonin treatment. In the treated animals, areas of necrosis were often associated with large amounts of predominantly mononuclear inflammatory infiltrate, which suggests that melatonin likely induces Th1 immune responses, thereby containing the infection and reducing the size of liver abscesses.

Amoebae-derived substances are known to be important factors for tissue damage [22-24]. Therefore, the reduction of necrotic areas may also be related to parasite factors, such as proteinases and amoebapores, which may be present in smaller proportions in the areas treated with melatonin, given the reduced presence of trophozoites in these regions.

It is worth mentioning that hamsters were chosen as an experimental infection model because they are more susceptible to infection by *E. histolytica *than mice, and criteria for determining the severity of their injuries are well established, thus allowing differences resulting from the administration of melatonin to be better distinguished. Our results indicate that there are beneficial effects of exogenous melatonin, corroborating reports that have evaluated melatonin as a modulator of other parasitic infections [16-21,25].

In a model of schistosomiasis in hamsters, melatonin increases the efficacy of cercarial and soluble worm antigens in inducing protective immunity against infection, suggesting possible applications in a vaccination program [26]. Melatonin can synchronize the cycle of the malaria parasite; the rupture of infected erythrocytes coincides with the timed release of melatonin in vertebrates, which occurs between midnight and three a.m. [27].

Phagocytosis plays an important role in *E. histolytica *pathogenicity, making it an object of investigations. A comprehensive evaluation of the phagocytic capacity of amoebae involves leukophagocytosis because amoebae are constantly in contact with leukocytes *in vivo *and must be able to destroy them to survive.

The identification of substances involved in leukocyte activation during amoeba-leukocyte interactions would help to direct the appropriate therapeutic use of these substances in amoebiasis. In this context, we evaluated the action of melatonin, a known immunomodulator, during PMN and MN leukophagocytosis performed by *E. histolytica *trophozoites.

Classical studies involving amoebae and leukocytes [28,29] have shown that virulent strains of amoebae are lethal for leukocytes, which lose their motility and are then phagocytosed and killed by the amoebae. In this study, we observed an increase in the rate of leukophagocytosis in the presence of the melatonin; however, this was accompanied by significantly higher rates of dead amoebae during the process. This suggests that melatonin plays a beneficial role for the host by activating PMN and MN cells. In addition, higher levels of superoxide anion and superoxide dismutase (SOD) were obtained during interactions in the presence of melatonin, which correlates with higher microbicidal leukocyte activity. Increased SOD may reflect antioxidant properties of melatonin which may be related to the reduced tissue damage.

These results suggest the need for further studies aiming to elucidate the mechanism of melatonin action in the host immune response. It would also be interesting to evaluate whether associations with other drugs may enhance the beneficial effects of melatonin in amoebiasis, as has been demonstrated for infections with *Trypanosoma cruzi *[30].

## Conclusions

The reduction in the areas of necrosis observed in experimental amoebic infection of hamsters and rats, together with the increased death of amoebae during leukophagocytosis, suggests that melatonin may play a beneficial role in the control of amoebic lesions and indicates that, as in other diseases, melatonin may be useful as an adjuvant in anti-amoebic therapy.

## Methods

All procedures were evaluated and institutionally approved by the Ethics Committee (protocol 6815/41), in compliance with the Helsinki Declaration and international guidelines for experimental research on animals.

### *In vivo *experiments

Trophozoites of the virulent strain of *E. histolytica *HM1:IMSS were grown axenically in a TYI-S-33 medium [31]. Parasites were maintained with thrice-weekly sub-cultures, assuring their use in the exponential growth phase.

Amoebas from axenic cultures were centrifuged at 200 × g in individual tubes, washed twice with PBS (pH 7.2) and adjusted to 1 × 10^6 ^amoebae in 0.1 mL of inoculum.

Six hamsters (*Mesocricetus auratus*), intestinal amoebiasis model and and six Wistar rats (*Rattus norvegicus*) amoebic liver model [32] were used for each group of experiments. In the case of both species the animals were male and approximately 40 days old, with the hamsters weighing around 60 g and the rats weighing around 110 g.

The animals were anesthetized with sodium pentobarbital (30 mg/kg for rats and 79 mg/kg for hamsters) and laparotomised, and the amoebae were inoculated directly into the liver (hamsters) or caecum (Wistar rats). Six days after inoculation, animals were sacrificed and dissected for macroscopic examination of the liver or caecum. Fragments of hamster livers and rat caeca were fixed in 10% buffered formalin, pH 7.2. After the fixation period, the fragments were dehydrated, diaphanised,[what is diaphanised? Do you mean cleared?] infiltrated and embedded in paraffin. Sections obtained for staining with hematoxylin and eosin (H&E) were 4 mm [not mm surely??]thick.

Areas of amoebic liver necrosis were quantified using the KS300 software contained in the Carl Zeiss image analyser. For the evaluation of necrosis, the sections were examined "in totum" and all images of destroyed liver parenchyma were digitalised using a 4× or 10× objective lens and a JVC TK-1270/RGB micro camera. The caeca were examined under an optical microscope to assess the presence or absence of amoebic lesions.

The inoculated animals were treated with melatonin (Sigma, St. Louis, MO, USA) subcutaneously [33] at a dose of 15 mg/kg body weight [34]. The drug was dissolved in deionised water and given once a day during the six days of the experiment. Treatment was started on the same day as the infection. All procedures for handling melatonin occurred in a darkened room.

### *In vitro *experiments

After obtaining written consent, heparinised blood was obtained from 5 healthy male volunteers aged 18-36 years old. Each sample comprised of approximately 10 ml of blood. A total of 15 samples (3 samples from each donor) were collected in order to complete the different experiments.

The heparinised blood was fractioned by centrifugation with Ficoll-Paque (Pharmacia, Upsala, Sweden) and subjected to sedimentation with dextran. This separation resulted in the formation of a PMN leukocyte sediment and an MN leucocyte layer at the Ficoll-Paque interface. This procedure resulted in preparations of 95% pure MN and PMN cells. MN and PMN cells were resuspended in PBS (*phosphate buffered saline - pH 7.4*) and washed. Resulting suspensions were adjusted to a concentration of 2 × 10^6 ^cells/mL [35].

One millilitre suspensions comprising leukocytes (2 × 106/mL) and amoebae (1 × 106/mL) in PBS were placed in 15 mL Falcon tubes. The tubes were briefly filled to 95% of their capacity with PBS and each suspension of leucocytes (either MN or PMN) and amoebae was then pelleted and resuspended in 1 mL of PBS. The tubes were then shaken at 37°C for 60 minutes to allow the leucocytes to interact with the trophozoites. Assays with the MN and PMN cells were adapted from a previously published protocol [35]. To evaluate possible melatonin effects on amoebae and leukocytes, the drug was added to the incubation medium at a concentration of 100 ng/mL. This is considered to be a pharmacological dose [36] and is generally used to evaluate cellular activation [37].

Phagocytosis was interrupted by incubating the tubes on ice for 10 min. The leukocytes and trophozoites were then stained with 200 μl of acridine orange (14.4 mg/L) for two minutes and washed twice in ice-cold PBS [38]. Indices of adherence, ingestion and death of the trophozoites and leukocytes were determined by fluorescence microscopy (TIM-4000, Germany) and compared with controls performed under the same conditions without the addition of melatonin. One hundred amoebae were counted per slide.

Superoxide release (O_2_^-^) was measured by reducing cytochrome C (Sigma) as previously described [39] and in order to determine the concentration of the enzyme superoxide dismutase (SOD), the adapted protocol [40].

An unpaired t test was used to analyse differences in leukophagocytosis between the two groups. Differences were considered to be statistically significant when the p value was lower than 0.05.

## Competing interests

The authors declare that they have no competing interests.

## Authors' contributions

ACFB and MAG conceived the study. FMSO, MVC and MAG carried out the experiments *in vivo*. ACFB, JLF, ELF and ACHF carried out the experiments *in vitro*. All authors participated in the study design, analysis of the results, drafted the manuscript and have given final approval of the version to be published.
